# Risk-Adapted Postmastectomy Radiotherapy Decision Based on Prognostic Nomogram for pT1-2N1M0 Breast Cancer: A Multicenter Study

**DOI:** 10.3389/fonc.2020.588859

**Published:** 2020-12-11

**Authors:** Ming Li, Jinbo Yue, Xiangbo Wan, Bin Hua, Qiuan Yang, Pei Yang, Zijian Zhang, Qian Pei, Weidong Han, Yaping Xu, Xuefeng Xia

**Affiliations:** ^1^ Department of Radiation Oncology, Beijing Hospital/National Center of Gerontology, Beijing, China; ^2^ Department of Radiation Oncology, Shandong Cancer Hospital Affiliated to Shandong University/Shandong Academy of Medical Sciences, Jinan, China; ^3^ Department of Radiation Oncology, The Sixth Affiliated Hospital - Sun Yat-sen University, Guangzhou, China; ^4^ Department of Breast Cancer Surgery, Beijing Hospital/National Center of Gerontology, Beijing, China; ^5^ Department of Radiation Oncology, Qilu Hospital of Shandong University, Jinan, China; ^6^ Department of Radiation Oncology, Hunan Cancer Hospital/The Affiliated Cancer Hospital of Xiangya School of Medicine - Central South University, Changsha, China; ^7^ Department of Radiation Oncology, Xiangya Hospital - Central South University, Changsha, China; ^8^ Department of General Surgery, Xiangya Hospital - Central South University, Changsha, China; ^9^ Department of Medical Oncology, Sir Run Run Shaw Hospital, College of Medicine, Zhejiang University, Hangzhou, China; ^10^ Geneplus-Beijing Institute, Beijing, China

**Keywords:** breast neoplasms, mastectomy, radiation therapy, nomogram, risk-adapted therapy, molecular subtype, prognosis, recurrence

## Abstract

**Purpose:**

The aim of this study was to develop a widely accepted prognostic nomogram and establish a risk-adapted PMRT strategy based on locoregional recurrence for pT1-2N1M0 breast cancer.

**Methods and Materials:**

A total of 3,033 patients with pT1-2N1M0 breast cancer treated at 6 participating institutions between 2000 and 2016 were retrospectively reviewed. A nomogram was developed to predicted locoregional recurrence-free survival (LRFS). A propensity score-matched (PSM) analyses was performed in risk-adapted model.

**Results:**

With the median follow-up of 65.0 months, the 5-year overall survival (OS), disease free survival (DFS) and LRFS were 93.0, 84.8, and 93.6%, respectively. There was no significant difference between patients who received PMRT or not for the entire group. A nomogram was developed and validated to estimate the probability of 5-year LRFS based on five independent factors including age, primary tumor site, positive lymph nodes number, pathological T stage, and molecular subtype that were selected by a multivariate analysis of patients who did not receive PMRT in the primary cohort. According to the total nomogram risk scores, the entire patients were classified into low- (40.0%), moderate- (42.4%), and high-risk group (17.6%). The 5-year outcomes were significantly different among these three groups (P<0.001). In low-risk group, patients who received PMRT or not both achieved a favorable OS, DFS, and LRFS. In moderate-risk group, no differences in OS, DFS, and LRFS were observed between PMRT and no PMRT patients. In high-risk group, compared with no PMRT, PMRT resulted in significantly different OS (86.8 vs 83.9%, P = 0.050), DFS (77.2 vs 70.9%, P = 0.049), and LRFS (90.8 vs. 81.6%, P = 0.003). After PSM adjustment, there were no significant differences in OS, DFS, and LRFS in low-risk and moderate-risk groups. However, in the high-risk group, PMRT still resulted in significantly better OS, DFS and improved LRFS.

**Conclusions:**

The proposed nomogram provides an individualized risk estimate of LRFS in patients with pT1-2N1M0 breast cancer. Risk-adapted PMRT for high-risk patients is a viable effective strategy.

## Introduction

In the past 3 decades, breast cancer has been recognized as a heterogeneous clinicopathological course. Primary tumor size (T stage) and number of axillary lymph nodes metastasis (N stage) are the most important factors affecting the locoregional recurrence and survival rate (1). Postmastectomy radiotherapy (PMRT) has been demonstrated to bring significant clinical benefits for high-risk patients in locoregional recurrence after mastectomy and systemic treatments (2–5). Relevant guidelines or consensus recommend that PMRT should be delivered for patients with tumor size more than 5 cm or with 4 or more axillary positive lymph nodes (stage T3-4 or N2-3), - infiltration of the skin, and/or the pectoral muscle, inflammatory carcinoma and positive margins. Patients with 1–3 positive axillary lymph nodes of early stage breast cancer (stage T1-2N1) have locoregional recurrence rate (LRR) of approximately 10–20% after mastectomy. Multiple retrospective analyses or subgroup analyses have highlighted the clinical benefit of PMRT for N1 patients (6–13). The results of Early Breast Cancer Trialists’ Collaborative Group (EBCTCG) meta-analysis showed that PMRT after mastectomy and axillary node dissection reduced both LRR and breast cancer mortality in patients with N1 stage breast cancer even when systemic therapy was administered (14). But due to the lack of randomized controlled and large-sample studies, the optimal therapy for N1 patients is still equivocal. It is not clear whether these patients need radiotherapy. Whether patients with T1-2N1 breast cancer should be treated with PMRT is also controversial, and there were no direct evidences to support the application of PMRT in T1-2N1 patients.

The 2018 ATTM meeting also presented that PMRT might be safely omitted in some early stage with good prognostic features breast cancer patients who received PST, and attempts were made to identify such patients. As recommendations were not uniform, doubts persist about which candidates can safely omit PMRT. The ATTM suggested that biomarker assessments might improve understanding of breast cancer biology and behavior (15).

In this study, we develop a widely accepted prognostic nomogram for the estimation of LRR for pT1-2N1M0 breast cancer. Then we stratified the patients into different risk categories based on the total nomogram risk scores, compare the efficacy of PMRT in different risk stratification, and finally optimize a risk-adapted therapeutic strategy.

## Methods and Materials

### Patient Eligibility

A total of 3,033 female patients with previously untreated infiltrating breast cancer with T1,2 disease and 1 to 3 positive lymph nodes who were treated with mastectomy followed by adjuvant systemic therapy at 6 participating institutions between 2000 and 2016 were retrospectively reviewed. To develop a nomogram prognostic model, all patients were divided into 2 cohorts. The primary cohort comprised 2031 patients from 4 institutions, and the validation cohort consisted of an independent series of 1,002 patients from the rest 2 institutions. All patients had complete clinical information and underwent standard staging procedures. Other eligibility requirements included typical histological and immunophenotypic features of breast cancer (World Health Organization (WHO) classification), pT1-2N1M0 disease (American Joint Committee on Cancer (AJCC) staging system), and complete follow-up information. Patients who had bilateral breast cancer or other malignances before or meanwhile or less than 1 year follow up time were excluded. This project was approved by the ethics committee at the Beijing Hospital/National Center of Gerontology and conducted in accordance with the Helsinki declaration of the World Medical Association (the 5^th^ revision in October 2000).

One of the variables adequately evaluated was the phenotype of immunohistochemical (IHC) receptor [including estrogen receptor (ER), progesterone receptor (PR), Human epidermal growth factor receptor 2 (Her2), and Antigen identified by monoclonal antibody Ki-67 (Ki-67)], which allowed us to categorize patients into 4 molecular subtypes (St. Gallen consensus 2013) as Luminal A (ER+ or PR+, HER2-), Luminal B (ER+ or PR+, HER2+), Her2 enriched (ER-, PR-, HER2+), and Triple negative (ER-, PR-, HER2-).

### Treatment

There were 851 (28.1%) patients received PMRT, and 2182 (71.9%) without PMRT. Target of PMRT included chest wall with infraclavicular (axillary level III) and supraclavicular fields at a median dose of 50Gy (range, 46–50.4Gy; dose per fraction, 1.8–2Gy). Internal mammary nodes were irradiated when the tumor located in inner and central region.

All patients received breast cancer mastectomy with negative surgical margin. There are 95.8% patients with sufficient lymph node dissection that was defined as at least 10 lymph nodes removed. The median axillary lymph node numbers of dissection were 20. All patients received CT, among which 553 (18.2%) received cyclophosphamide-doxorubicin-fluorouracil (CAF) or adriamycin-contained regimens, whereas 2,480 (81.8%) received taxanes–based regimens. The number of CT cycles ranged from 6 to 8. There were 2,150 patients received at least 5 years of endocrine treatment, which accounted for 92.0% of all hormone receptor-positive patients. Among them, 973 (45.3%) patients were treated with aromatase inhibitors (AI). There were 617 (20.3%) patients had known HER2 positive status and among which 171 (27.7%) were treated with trastuzumab. The median lasting time of all targeted treatments was 12 months.

### Statistical Analyses

The primary endpoint of this study was locoregional recurrence-free survival (LRFS) as calculated from the start of initial surgical treatment until the time of locoregional recurrence, or until the last follow-up. LRR was defined as tumor recurrence in the ipsilateral chest wall, and supraclavicular, axillary or internal mammary lymph nodes. Secondary endpoints were disease-free survival (DFS) and overall survival (OS).

Survival curves were estimated with the Kaplan–Meier method and compared with a log-rank test stratified according to the prognostic factors. Cox proportional hazards regression model was performed to identify independent risk factors for LRFS in the primary cohort. The nomogram was formulated based on the Cox model parameter estimates. There were several steps to validate the efficacy of the nomogram. First, an internal validation was undertaken with a concordance index (C-index) being estimated by analyzing the area under the curve (AUC) of the receiver operating characteristic (ROC) curve. Next, a calibration plot was constructed by comparing the decile of predicted probabilities and actual probabilities using 1,000 bootstrap resamples. Finally, in external validation, the nomogram was used to assess each patient in the validation cohort, and the regression analysis was then used to derive the C-index and the calibration curve. Propensity score–matched (PSM) analysis was conducted to mirror randomized study design and generate comparable study arms; 1:1 patient matching without replacement was used to pair each patient receiving PMRT with another without PMRT whose propensity score was within the designated caliper size (in low-risk group, the ratio is 1:2). After PSM, baseline covariates and survival rates were compared between treatment groups. OS, DFS, LRFS were assessed with the Kaplan-Meier method, and compared using the log-rank test.

Cox proportional hazards regression was performed with IBM SPSS Statistics, version 25.0. Nomogram construction and validation were performed with Iasonos’ guide. Nomogram and bootstrap resampling were performed using the Hmisc, rms, survivalROC package in R, version 3.5.3 (http://www.r-project.org/). PSM was performed with Stata 15. Other analyses were performed with IBM SPSS Statistics 25.0. A 2-sided P values of less than 0.05 were considered significant.

## Results

### Patient Characteristics

Of all patients, the median age was 50.00 years (range, 21–84). Molecular subtype was presented as Luminal A (64.7%), Luminal B (12.4%), Her2 enriched (7.9%), and Triple negative (14.9%) types in all patients. The patients’ characteristics are presented in **Table 1**. Similar clinical characteristics were observed in the primary and the validation cohorts.

**Table 1:** Clinical characteristics of patients with pT1N1M0 breast cancer.

Characteristic	All patientsNo. (%)	Primary cohort	Validation cohort	P value	PMRT cohort	No PMRT cohort	P value
		No. (%)	No. (%)		No. (%)	No. (%)	
Total	3,033 (100)	2,031 (67.0)	1,002 (33.0)		851 (28.1)	2,182 (71.9)	
Lateral				0.601			0.793
left	1,537 (50.7)	1,036 (51.0)	501 (50.0)		428 (50.3)	1,109 (50.8)	
right	1,496 (49.3)	995 (49.0)	501 (50.0)		423 (49.7)	1,073 (49.2)	
Age (y)				0.339			< 0.001
≤ 45	982 (32.4)	646 (31.8)	336 (33.5)		351 (41.2)	631 (28.9)	
> 45	2,051 (67.6)	1,385 (68.2)	666 (66.5)		500 (58.8)	1,551 (71.1)	
Menstrual status				0.325			< 0.001
premenopausal	1,687 (55.6)	1,117 (55.0)	570 (56.9)		533 (62.6)	1,154 (52.9)	
postmenopausal	1,346 (44.4)	914 (45.0)	432 (43.1)		318 (37.4)	1,028 (47.1)	
Coronary disease				0.087			0.530
No	2,937 (98.0)	1,997 (98.3)	976 (97.4)		832 (97.8)	2,141 (98.1)	
Yes	60 (2.0)	34 (1.7)	26 (2.6)		19 (2.2)	41 (1.9)	
Primary tumor site				0.304			0.025
lateral	2,248 (74.1)	1,517 (74.7)	731 (73.0)		655 (77.0)	1,593 (73.0)	
Inner+central	785 (25.9)	514 (25.3)	271 (27.0)		196 (23.0)	589 (27.0)	
Grade				0.883			0.014
I	107 (3.5)	73 (3.6)	34 (3.4)		19 (2.2)	88 (4.0)	
II	2,123 (70.0)	1,416 (69.7)	707 (70.6)		586 (68.9)	1,537 (70.4)	
III	803 (26.5)	542 (26.7)	261 (26.0)		246 (28.9)	557 (25.5)	
Pathological MVI				0.729			< 0.001
Yes	314 (10.4)	213 (10.5)	101 (10.1)		114 (13.4)	200 (9.2)	
No	2,719 (89.6)	1,818 (89.5)	901 (89.9)		737 (86.6)	1,982 (90.8)	
Pathological LVI				0.399			0.172
Yes	19 (0.6)	11 (0.5)	8 (0.8)		8 (0.9)	11 (0.5)	
No	3,014 (99.4)	2,020 (99.5)	994 (99.2)		843 (99.1)	2,171 (99.5)	
Positive LN number				0.965			< 0.001
1	1,472 (48.5)	984 (48.4)	488 (48.7)		264 (31.0)	1,208 (55.4)	
2	935 (30.8)	625 (30.8)	310 (30.9)		274 (32.2)	661 (30.3)	
3	626 (20.6)	422 (20.8)	204 (20.4)		313 (36.8)	313 (14.3)	
Positive LN percentage				0.851			< 0.001
≤ 10%	1,905 (62.8)	1,278 (62.9)	627 (62.6)		390 (45.8)	1,515 (69.4)	
> 10%	1,128 (37.2)	753 (37.1)	375 (37.4)		461 (54.2)	667 (30.6)	
Pathological T stage				0.509			< 0.001
T1	1,412 (46.6)	937 (46.1)	475 (47.4)		345 (40.5)	1,067 (48.9)	
T2	1,621 (53.4)	1,094 (53.9)	527 (52.6)		506 (59.5)	1,115 (51.1)	
Molecular subtype				0.366			< 0.001
Luminal A	1,963 (64.7)	1,304 (64.2)	659 (65.8)		490 (57.6)	1,473 (67.5)	
Luminal B	376 (12.4)	244 (12.0)	132 (13.2)		115 (13.5)	261 (12.0)	
Her2 enriched	241 (7.9)	167 (8.2)	74 (7.4)		87 (10.2)	154 (7.1)	
Triple negative	453 (14.9)	316 (15.6)	137 (13.7)		159 (18.7)	294 (13.5)	
ER				0.009			< 0.001
Negative	892 (29.4)	628 (30.9)	264 (26.3)		303 (35.6)	589 (27.0)	
Positive	2,141 (70.6)	1,403 (69.1)	738 (73.7)		548 (64.4)	1,593 (73.0)	
PR				0.167			< 0.001
Negative	937 (30.9)	644 (31.7)	293 (29.2)		327 (38.4)	610 (28.0)	
Positive	2,096 (69.1)	1,387 (68.3)	709 (70.8)		524 (61.6)	1,572 (72.0)	
Her2				0.836			0.004
Negative	2,416 (79.7)	1,620 (79.8)	796 (79.4)		649 (76.3)	1,767 (81.0)	
Positive	617 (20.3)	411 (20.2)	206 (20.6)		202 (23.7)	415 (19.0)	
Ki-67				0.653			< 0.001
< 30%	953 (31.4)	628 (30.9)	325 (32.4)		253 (29.7)	700 (32.1)	
≥ 30%	585 (19.3)	391 (19.3)	194 (19.4)		206 (24.2)	379 (17.4)	
unknown	1,495 (49.3)	1,012 (49.8)	483 (48.2)		392 (46.1)	1,103 (50.5)	

MVI, microvascular invasion; LVI, lymphatic vessel invasion; ER, estrogen receptor; PR, progesterone receptor; Her2, Human epidermal growth factor receptor 2; Ki-67, Antigen identified by monoclonal antibody Ki-67; PMRT, Postmastectomy Radiotherapy.

### Construction and Internal Validation of the Nomogram

We identified clinical features that have previously been demonstrated to be associated with survival, and used univariate analysis to explore the prognostic features of patients who did not receive PMRT in primary cohort. The prognostic factors that predicted poor LRFS included age ≤45 years, premenopausal status, inner and central primary disease site, T2 stage, 2–3 positive lymph nodes, positive lymph nodes percentage >10%, ER (-), PR (-), Ki-67 ≥30%, and Her2 enriched or Triple negative molecular subtype (**Table 2**).

**Table 2:** Univariate analysis of the association between clinicopathological features and locoregional recurrence-free survival (LRFS) for patients with pT1N1M0 breast cancer in the primary cohort.

Characteristic	Primary cohort	
	5-year LRFS (%)	P value
Lateral		0.396
Left	93.0	
Right	92.9	
Age(y)		0.009
≤ 45	90.6	
> 45	93.8	
Menstrual status		0.030
premenopausal	91.7	
postmenopausal	94.2	
Coronary disease		0.364
No	93.2	
Yes	79.7	
Primary tumor site		0.004
Lateral	93.8	
Inner+central	90.6	
Grade		0.380
I	98.3	
II	93.0	
III	91.9	
Pathological MVI		0.141
Yes	93.4	
No	88.8	
Pathological LVI		0.538
Yes	92.9	
No	100	
Positive LN number		0.001
1	94.4	
2	93.3	
3	86.5	
Positive LN percentage		0.002
≤ 10%	94.3	
> 10%	89.8	
Pathological T stage		0.001
T1	95.8	
T2	90.3	
Molecular subtype		< 0.001
Luminal A	95.6	
Luminal B	91.0	
Her2 enriched	87.1	
Triple negative	84.3	
ER		< 0.001
Negative	86.7	
Positive	95.5	
PR		< 0.001
Negative	86.4	
Positive	95.7	
Her2		0.354
Negative	93.7	
Positive	89.5	
Ki-67		0.001
< 30%	95.8	
≥ 30%	92.6	
Unknown	91.4	

MVI, microvascular invasion; LVI, lymphatic vessel invasion; ER, estrogen receptor; PR, progesterone receptor; Her2, Human epidermal growth factor receptor 2; Ki-67, Antigen identified by monoclonal antibody Ki-67; PMRT, Postmastectomy Radiotherapy.

Multivariate analysis demonstrated that age (≤45 years vs. >45 years), primary tumor site (lateral region vs. inner and central region), molecular subtype (Luminal A vs. Luminal B vs. Her2 enriched vs. Triple negative), pathological T stage (T1 vs. T2) and positive lymph nodes number (1 vs. 2 vs. 3) were independent factors for LRFS (**Table 3**).

**Table 3:** Multivariate analysis of the association between clinicopathological features and locoregional recurrence-free survival (LRFS) for patients with pT1N1M0 breast cancer in the primary cohort.

Variable	LRFS		
	HR	95%CI	P value
Age (≤ 45y vs. > 45y)	1.692	1.144–2.504	0.008
Primary tumor site (Inner+central vs. Lateral)	2.013	1.365–2.969	< 0.001
Pathological T stage (T2 vs. T1)	1.942	1.294–2.915	0.001
Positive LN number			0.041
1	Ref.	Ref.	Ref.
2	1.569	0.934–2.634	0.089
3	2.310	1.205–4.427	0.012
Molecular subtype			< 0.001
Luminal A	Ref.	Ref.	Ref.
Luminal B	1.077	0.549–2.114	0.829
Her2 enriched	2.261	1.227–4.167	0.009
Triple negative	2.675	1.693–4.225	< 0.001

HR, Hazard Ratio; CI, confidence interval; LRFS, locoregional recurrence-free survival.

**Figure 1:**
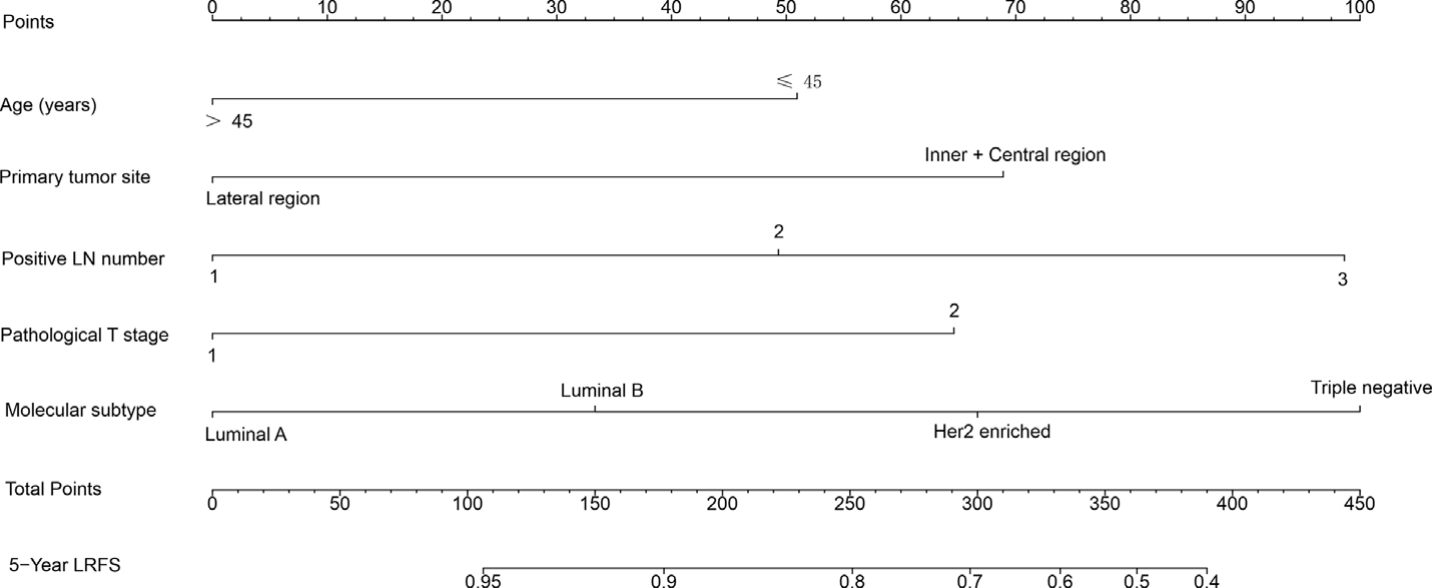
Nomogram predicting 5-year locoregional recurrence-free survival (LRFS) for patients with pT1-2N1M0 breast cancer. The nomogram assigns a point to each variable value according to its contributions. The sum of these numbers is located on the total points axis and can be translated to predicted probability of LRFS for a patient.

**Figure 2:**
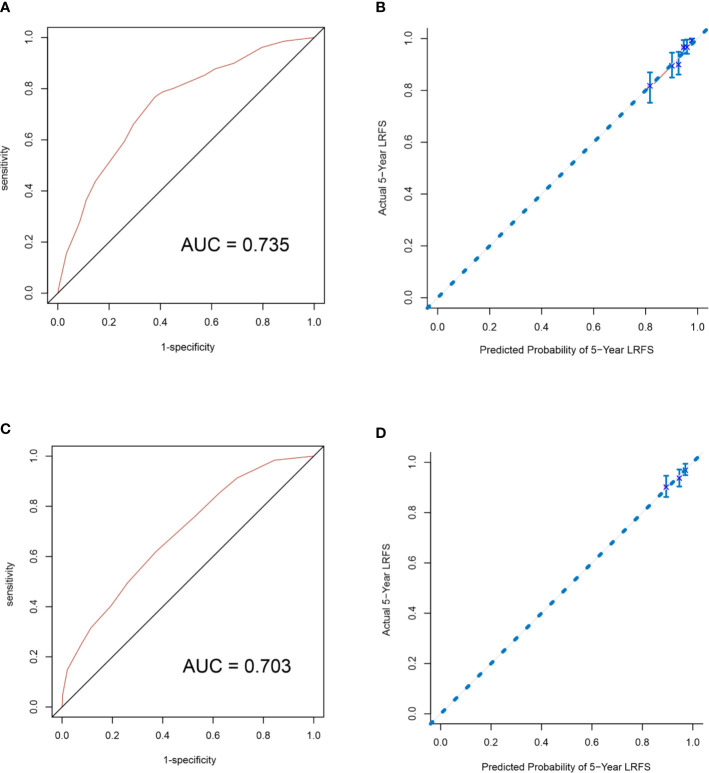
Internal validation of the nomogram to predict LRFS likelihoods in the primary cohort patients. The area under the receiver operating characteristic (ROC) curve (AUC) was 0.735 **(A)**. The calibration curve for the prediction of 5-year LRFS **(B)**. External validation of the nomogram to predict LRFS likelihoods in the validation cohort patients. The area under the receiver operating characteristic (ROC) curve (AUC) was 0.703 **(C)**. The calibration curve for the prediction of 5-year LRFS **(D)**.

A nomogram to predict 5-year LRFS was developed using the prognostic factors from the multivariate analysis (**Figure 1**). The predictive accuracy for 5-year LRFS was measured by the C-index was 0.735 in the internal validation (**Figure 2A**). The calibration plot for the probability of 5-year LRFS showed a good correlation between the actual observed outcome and the prediction by the nomogram (**Figure 2B**).

### External Validation of Nomogram for Locoregional Recurrence-Free Survival

The nomogram was validated to assess each patient who did not receive PMRT in the validation cohort. The C-index of the nomogram for the prediction of the 5-year LRFS was 0.703 in the external validation step (**Figure 2C**), which demonstrated that it is a model with a good level of discriminative ability. The calibration curve revealed that the nomogram was well calibrated; the 5-year LRFS showed an optimal agreement between the actual observation and the nomogram prediction (**Figure 2D**).

### Locoregional Recurrence Risk Stratification and Survival

**Figure 3:**
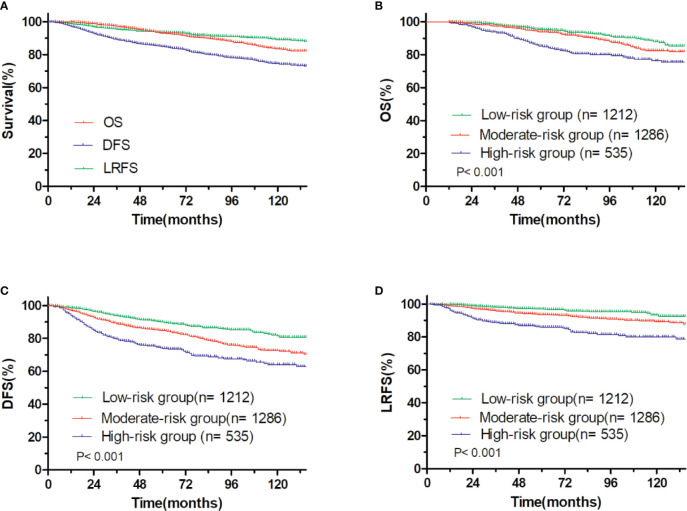
OS and DFS and LRFS for all patients. **(A)** pT1-2N1M0 breast cancer, **(B)** OS, and **(C)** DFS, and **(D)** LRFS for patients with pT1-2N1M0 breast cancer stratified into the low- and moderate- and high-risk groups.

At a median follow up time of 65.00 months (95% *CI*: 62.96–67.04 months), the 5-year OS, DFS and LRFS were 93.0, 84.8, and 93.6%, respectively (**Figure 3A**). There were 215 patients had locoregional recurrence events, including 107 (49.8%) in ipsilateral chest wall, 42 (19.5%) in axilla lymph nodes, 120 (55.8%) in supraclavicular lymph nodes, and 36 (16.7%) in internal mammary lymph nodes. According to the total nomogram risk scores of the patients to estimate the probability of 5-year LRFS, the cohort was stratified into three groups (≤100, low-risk, 40.0%; 101–199, moderate-risk, 42.4%; ≥200, high-risk, 17.6%) representing distinct prognosis. The 5-year outcomes were significantly different among the three groups, with 5-year OS, DFS, and LRFS rates of 95.5, 89.8, and 96.9% for the low-risk group, 93.8, 84.7, and 93.5% for the moderate-risk group and 85.2% (P<0.001, **Figure 3B**), 73.8% (P<0.001, **Figure 3C**), and 85.8% (P<0.001, **Figure 3D**) for the high-risk group.

### PMRT Showed Limited Benefit in Low- and Moderate-Risk Group, but Improved Survivals in High-Risk Group

First, we evaluated the efficacy of PMRT vs. without PMRT. In the unadjusted population, patients treated with PMRT tended to have more risk factors than those without PMRT (**Table 1**). But they both achieved a good outcome. For all patients, no effective difference was found between with PMRT and without PMRT (5-year OS, 93.3 vs. 92.2%, P = 0.256; 5-year DFS, 82.3 vs. 85.8%, P = 0.088; 5-year LRFS, 94.3 vs. 93.2%, P = 0.360).

**Figure 4:**
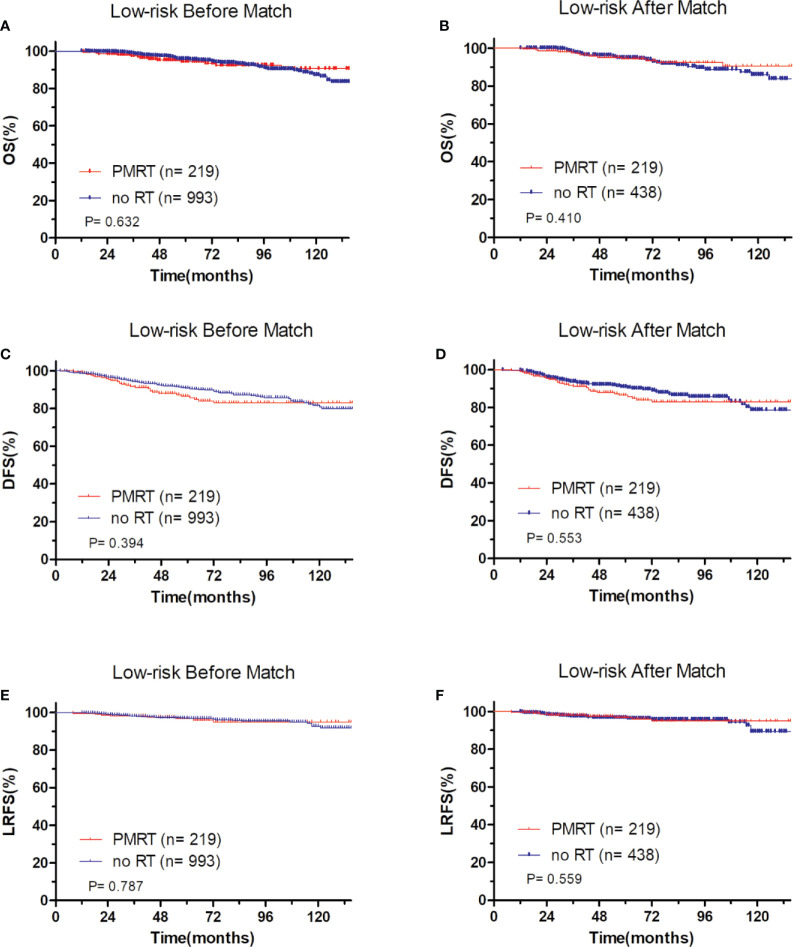
Comparison of OS and DFS and LRFS between with PMRT and without PMRT for low-risk pT1-2N1M0 patients. For patients with PMRT or without PMRT, OS before **(A)** and after **(B)** match stratification; DFS before **(C)** and after **(D)** match stratification; LRFS before **(E)** and after **(F)** match stratification.

**Figure 5:**
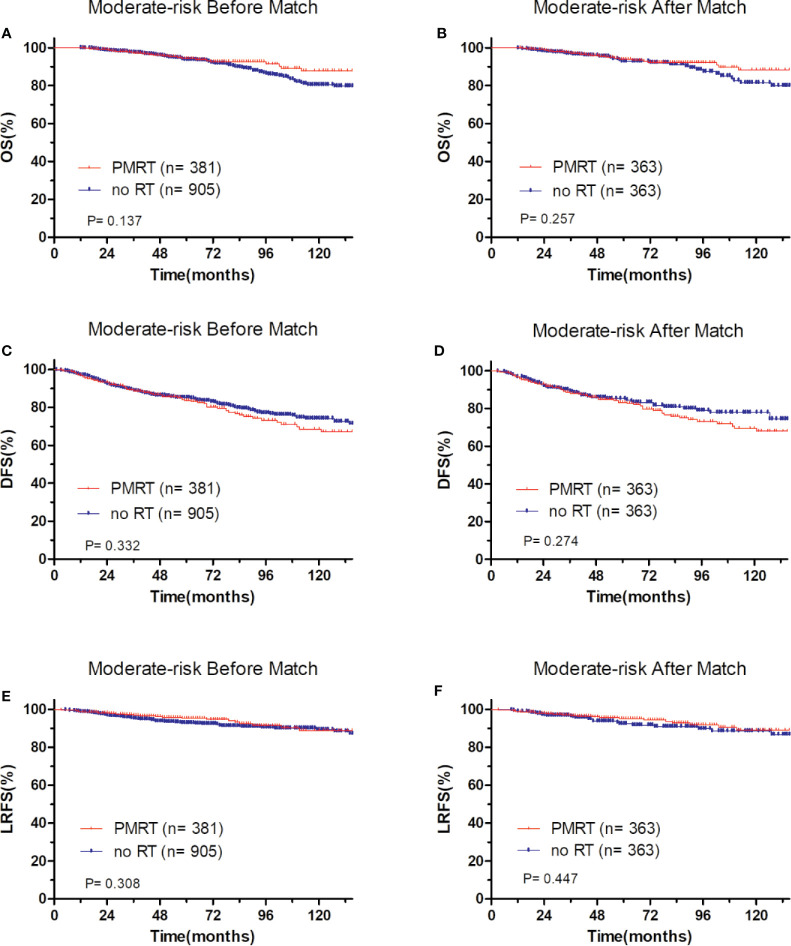
Comparison of OS and DFS and LRFS between with PMRT and without PMRT for moderate-risk pT1-2N1M0 patients. For patients with PMRT or without PMRT, OS before **(A)** and after **(B)** match stratification; DFS before **(C)** and after **(D)** match stratification; LRFS before **(E)** and after **(F)** match stratification.

**Figure 6:**
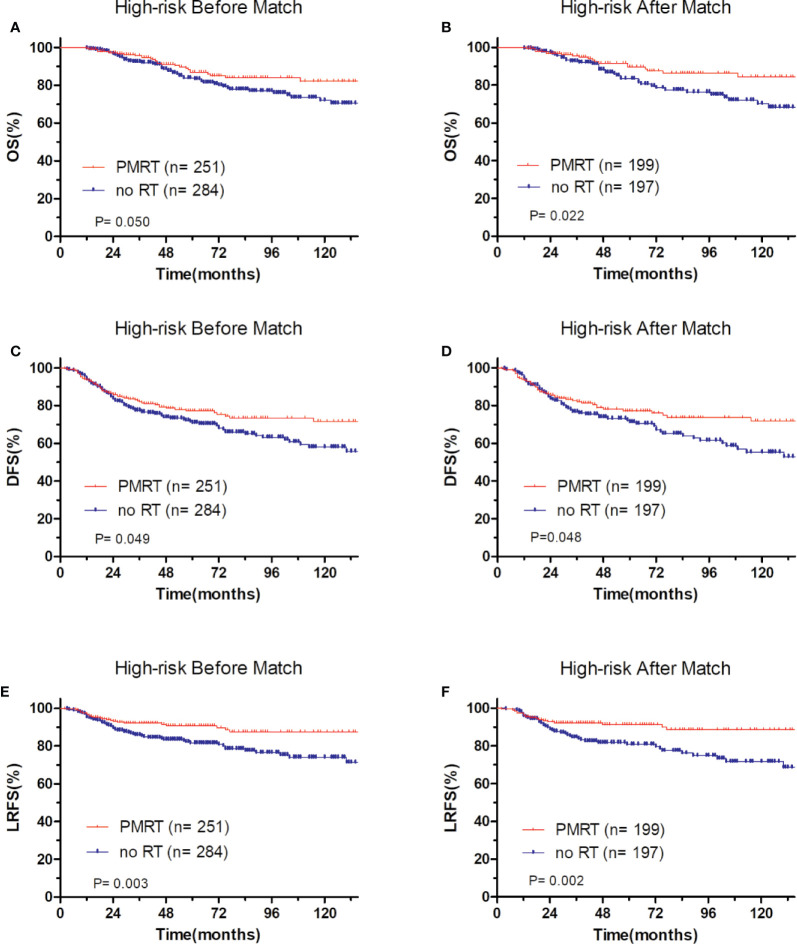
Comparison of OS and DFS and LRFS between with PMRT and without PMRT for high-risk pT1-2N1M0 patients. For patients with PMRT or without PMRT, OS before **(A)** and after **(B)** match stratification; DFS before **(C)** and after **(D)** match stratification; LRFS before **(E)** and after **(F)** match stratification.

In low-risk group, patients who received PMRT or not both achieved a favorable OS (94.4 vs. 95.8%, P = 0.632, **Figure 4A**), DFS (86.1 vs. 90.7%, P = 0.394, **Figure 4C**), and LRFS (96.7 vs. 97.0%, P = 0.787, **Figure 4E**). Similarly, in moderate-risk group, no differences were found in OS (94.3 vs. 93.7%, P = 0.137, **Figure 5A**), DFS (83.4 vs. 85.2%, P = 0.332, **Figure 5C**), and LRFS (95.3 vs. 92.8%, P = 0.308, **Figure 5E**) between PMRT or no PMRT patients. In high-risk group, compared with no PMRT, differences were observed in OS (86.8 vs. 83.9%, P = 0.050, **Figure 6A**), DFS (77.2 vs. 70.9%, P = 0.049, **Figure 6C**), and LRFS (90.8 vs. 81.6%, P = 0.003, **Figure 6E**) of PMRT patients.

After adjustment by PSM, the clinicopathological characteristics of patients in each cohort were balanced between treatment groups (**Table 4**–**6**). There were no significant differences in OS, DFS, and LRFS in low-risk group (**Figures 4B, D, F**) and moderate-risk group (**Figures 5B, D, F**). In the high-risk group, PMRT still resulted in significantly better OS (89.7 vs. 83.4%, P = 0.022, **Figure 6B**), DFS (77.1 vs. 71.3%, P = 0.048, **Figure 6D**), and LRFS (91.3 vs. 80.8%, P = 0.002, **Figure 6F**).

**Table 4:** Clinical characteristics of low-risk patients with pT1-2N1M0 breast cancer before and after PSM 1:2 stratification by treatment.

Characteristic	Before match			After match		
	PMRT (%)	No PMRT (%)	P value	PMRT (%)	No PMRT (%)	P value
Total	219	993	–	219	438	–
Age (y)			0.005			0.562
≤ 45	50 (22.8)	150 (15.1)		50 (22.8)	109 (24.9)	
> 45	169 (77.2)	843 (84.9)		169 (77.2)	329 (75.1)	
Primary tumor site			0.001			1.000
lateral	209 (95.4)	871 (87.7)		209 (95.4)	418 (95.4)	
Inner+ central	10 (4.6)	122 (12.3)		10 (4.6)	20 (4.6)	
Positive LN number			< 0.001			0.233
1	133 (60.7)	753 (75.8)		133 (60.7)	266 (60.7)	
2	53 (24.2)	192 (19.3)		53 (24.2)	124 (28.3)	
3	33 (15.1)	48 (4.8)		33 (15.1)	48 (11.0)	
Pathological T stage			0.939			1.000
T1	171 (78.1)	773 (77.8)		171 (78.1)	342 (78.1)	
T2	48 (21.9)	220 (22.2)		48 (21.9)	96 (21.9)	
Molecular subtype			0.942			0.902
Luminal A	180 (82.2)	814 (82.0)		180 (82.2)	351 (80.1)	
Luminal B	24 (11.0)	110 (11.1)		24 (11.0)	57 (13.0)	
Her2 enriched	5 (2.3)	29 (2.9)		5 (2.3)	10 (2.3)	
Triple negative	10 (4.6)	40 (4.0)		10 (4.6)	20 (4.6)	

PMRT, Postmastectomy Radiotherapy.

**Table 5:** Clinical characteristics of moderate-risk patients with pT1-2N1M0 breast cancer before and after PSM 1:1 stratification by treatment.

Characteristic	Before match			After match		
	PMRT (%)	No PMRT (%)	P value	PMRT (%)	No PMRT (%)	P value
Total	379	840	–	363	363	–
Age (y)			0.483			1.000
≤ 45	140 (36.7)	314 (34.7)		127 (35.0)	127 (35.0)	
> 45	241 (63.3)	591 (65.3)		236 (65.0)	236 (65.0)	
Primary tumor site			< 0.001			1.000
lateral	301 (79.0)	583 (64.4)		286 (78.8)	286 (78.8)	
Inner+ central	80 (21.0)	322 (35.6)		77 (21.2)	77 (21.2)	
Positive LN percentage			< 0.001			1.000
1	105 (27.6)	385 (42.5)		105 (28.9)	105 (28.9)	
2	152 (39.9)	373 (41.2)		152 (41.9)	152 (41.9)	
3	124 (32.5)	147 (16.2)		106 (29.2)	106 (29.2)	
Pathological T stage			0.037			1.000
T1	130 (34.1)	256 (28.3)		112 (30.9)	112 (30.9)	
T2	251 (65.9)	649 (71.7)		251 (69.1)	251 (69.1)	
Molecular subtype			0.164			1.000
Luminal A	225 (59.1)	584 (64.5)		212 (58.4)	212 (58.4)	
Luminal B	67 (17.6)	122 (13.5)		64 (17.6)	64 (17.6)	
Her2 enriched	47 (12.3)	96 (10.6)		45 (12.4)	45 (12.4)	
Triple negative	42 (11.0)	103 (11.4)		42 (11.6)	42 (11.6)	

PMRT, Postmastectomy Radiotherapy.

**Table 6:** Clinical characteristics of high-risk patients with pT1-2N1M0 breast cancer before and after PSM 1:1 stratification by treatment.

Characteristic	Before match			After match		
	PMRT (%)	No PMRT (%)	P value	PMRT (%)	No PMRT (%)	P value
Total	251	284	–	199	197	–
Age (y)			0.206			0.937
≤ 45	161 (64.1)	167 (58.8)		123 (61.8)	121 (61.4)	
> 45	90 (35.9)	117 (41.2)		76 (38.2)	76 (38.2)	
Primary tumor site			0.041			0.774
lateral	145 (57.8)	139 (48.9)		120 (60.3)	116 (58.9)	
Inner+ central	106 (42.2)	145 (51.1)		79 (39.7)	81 (41.1)	
Positive LN percentage			< 0.001			0.996
1	26 (10.4)	70 (24.6)		26 (13.1)	26 (13.2)	
2	69 (27.5)	96 (33.8)		64 (32.2)	64 (32.5)	
3	156 (62.2)	118 (41.5)		109 (54.8)	107 (54.3)	
Pathological T stage			0.184			0.739
T1	44 (17.5)	38 (13.4)		26 (13.1)	28 (14.2)	
T2	207 (82.5)	246 (86.6)		173 (86.9)	169 (85.8)	
Molecular subtype			0.067			0.966
Luminal A	85 (33.9)	75 (26.4)		65 (32.7)	64 (32.5)	
Luminal B	24 (9.6)	29 (10.2)		20 (10.1)	17 (8.6)	
Her2 enriched	35 (13.9)	29 (10.2)		22 (11.1)	23 (11.7)	
Triple negative	107 (42.6)	151 (53.2)		92 (46.2)	93 (47.2)	

PMRT, Postmastectomy Radiotherapy.

## Discussion

PMRT in T1-2N1 breast cancer is an important clinical study subject that has not been solved due to lack of randomized controlled trails. Our study is a multicenter retrospective study with large sample size, trying to explore the value of PMRT for pT1-2N1M0 breast cancer in the modern era. Our study showed that the overall LRR of pT1-2N1M0 breast cancer after mastectomy is low and the survival rate is high. This is consistent with the international advanced level. In multivariate analysis, age, primary tumor site, pT stage, number of positive lymph nodes, and molecular subtype were identified as independent risk factors for locoregional recurrence. We developed a nomogram to estimate the probability of 5-year LRFS based on these five variables. Stratified analysis based on the nomogram total risk scores showed that the high-risk group exhibited a high LRR, while the low-risk group had a locoregional recurrence risk <10%. PMRT significantly improved LRFS of patients in the high-risk group. To our knowledge, this is the first study to develop a pT1-2N1M0 breast cancer-specific nomogram based on a large cohort of patients. The nomogram has been validated as a reliable tool to predict survival in these patients, independent of treatment strategy.

We selected cases treated with systemic therapy since 2000, and data showed that the standards of screening, diagnosis, and treatment for breast cancer continue to improve over time. All patients received standard modified radical mastectomy. More than 80% patients received paclitaxel chemotherapy. Almost all ER or PR receptor-positive patients received endocrine therapy, among them, nearly half were treated with AI. In views of the economic factors, 1/3 of HER2-positive patients chose targeted therapy. Then, we divided the entire patients into two groups according to the different institutions. We constructed a prognostic model in four institutions, and observed that the established model still had good predictive efficacy in other two institutions. This led us to further stratify all patients into different risk groups on the nomogram scores.

Emerging evidences have shown that LRR of patients without lymph node metastasis (N0 stage) after mastectomy was less than 10%, and PMRT did not improve LRR. For patients with more than 4 positive lymph nodes, PMRT not only reduced the LRR, but also improved the OS. Meanwhile, the meta-analysis showed that PMRT significantly reduced 5 years LRR (from 16.5 to 3.8%) in breast cancer patients with 1-3 positive lymph nodes (N1 stage), and improved the 5, 10, and 20 years survival rates by 5.6, 9.9, and 7.9% (14). However, whether all T1-2N1M0 patients need PMRT remains controversial. Firstly, previous results were derived from early studies in which most patients received adriamycin or non-adriamycin chemotherapy without targeted therapy, and randomized trials in the 1980s and early 1990s reported LRRs of 12 to 30%. With the development of systemic therapy in recent years, taxol and targeted therapy further reduced the 10 years LRR to 10–13% in patients not received PMRT (16–18). Although the LRR is significantly lower than before, it is still possible that PMRT may be benefit for patients with this degree of LRR risk. A previous analysis of MDACC demonstrated that PMRT reduced the 10-year LRR risk from 13 to 3% (P = 0.003) (19). However, the long-term survival benefit brought by radiotherapy may be reduced. Secondly, there is heterogeneity in pT1-2N1M0 stage breast cancer patients, and the LRR of some low-risk patients is less than 10%. These patients may not need radiotherapy because the long-term survival benefit is very low. Modern changes in management have affected the benefits of PMRT, the reported LRR have further decreased (20–23). According to the latest data of MDACC and MSKCC, the risk of failure of patients treated after 2000 has decreased to about 4%. More recently, a study from MDACC retrospectively analyzed LRR in 1027 T1-2N1 patients during an early era (1978–1997) and a later era (2000–2007) (24). These eras were divided due to the routine use of standard surgery, taxane chemotherapy, and aromatase inhibitors. Results are similar to our study. In the later era, there are 25% of the 522 patients with significant higher-risk features received PMRT. PMRT did not appear to benefit these patients, with 5-year LRR of 2.8% for non-PMRT and 4.2% for PMRT (HR 1.41, P = 0.48).

With such low LRR in the modern era, PMRT may not benefit all patients with 1–3 positive lymph nodes. Rather than adopting a universal recommendation of PMRT for all N1 patients, many institutional philosophy has been to selectively recommend PMRT for subcategories of patients who have higher-risk features over the past decade. Clinicopathological factors are important factors affecting the LRR of breast cancer. The results of this study showed that we also used these risk factors to select patients for radiotherapy. Current treatment of breast cancer also uses biologic features into clinical decision making (25–29). Previous studies have shown that molecular subtype (MST) can predict recurrence and survival in breast cancer. HER2 enriched and triple negative subtypes has been shown to correlate with the poorest outcomes (30–36). However, there are limited reports on MST association with LRR in the N1 group. A study from MSKCC investigated the association of MST with LRR and PMRT effect among T1-2N1 patients (37). Results showed a trend toward an increase in LRR in the HER2 and basal subtypes (5.4 and 8.8%, respectively).

In this study, for the first time, we combined clinicopathological factors with molecular typing to establish a stratified model of prognostic risk, suggesting that the use of appropriate molecular markers may help better screen out patients at high risk of recurrence and avoid overtreatment. Age, site of primary lesion, molecular type, pT stage and positive lymph nodes number were independent factors affecting LRFS. Low-risk, moderate-risk, and high-risk patients had significantly different outcomes. PMRT reduced the LRR of patients in the high-risk group, and statistically significant improved the DFS rate and OS rate (38). However, related to the small number of patients and short follow-up time, whether PMRT can really improve the long-term survival of high-risk patients rely on additional validation with expanded sample size and long-term follow-up, and the results are expected to provide help for phase III clinical research design.

There are some limitations in this retrospective study. Because of the non-random assignment of treatments, high-risk patients were more likely to be selected to receive PMRT. Thus, the survival results may be affected by selection biases. To reduce the influence of this limitation, we used PSM to account for prognostic factors. The numbers of patients in each treatment group were sufficient to compare survival differences after PSM adjustment.

In conclusion, LRR after mastectomy of pT1-2N1M0 breast cancer is low, but we have developed and externally validated a nomogram that can predict 5-year LRFS with a high degree of accuracy based on a large cohort of patients. Risk-adapted PMRT for high-risk patients is a viable effective strategy based on the total nomogram risk scores. Because MST does predict survival outcomes, it should to be a useful tool to identify patients who would benefit from PMRT in this subgroup. Future prospective studies are required to refine.

## Data Availability Statement

The original contributions presented in the study are included in the article/supplementary material. Further inquiries can be directed to the corresponding author.

## Ethics Statement

This project was approved by the ethics committee at the Beijing Hospital/National Center of Gerontology and conducted in accordance with the Helsinki Declaration of the World Medical Association (the 5th revision in October 2000).

## Author Contributions

ML conceived and designed the experiments. ML, JY, and XW participated in the experiments and drafted the manuscript. ML, JY, XW, BH, QY, PY, ZZ, and QP contributed to the sample collection and interpretation of the data. ML, JY, XW, and WH performed the statistical analysis. BH, QY, PY, ZZ, QP, WH, YX, and XX revised the manuscript. All authors read and approved the final manuscript. All authors contributed to the article and approved the submitted version.

## Funding

This study was supported by a Beijing Hospital Grant (grant number, bj-2018-018) from Beijing Hospital/National Center of Gerontology.

## Conflict of Interest

Authors YX and XX were employed by company Geneplus-Beijing Institute.

The remaining authors declare that the research was conducted in the absence of any commercial or financial relationships that could be construed as a potential conflict of interest.
